# Analysis of circulating extracellular vesicle derived microRNAs in breast cancer patients with obesity: a potential role for Let-7a

**DOI:** 10.1186/s12967-023-04075-w

**Published:** 2023-03-31

**Authors:** Ines Barone, Luca Gelsomino, Felice Maria Accattatis, Francesca Giordano, Balazs Gyorffy, Salvatore Panza, Mario Giuliano, Bianca Maria Veneziani, Grazia Arpino, Carmine De Angelis, Pietro De Placido, Daniela Bonofiglio, Sebastiano Andò, Cinzia Giordano, Stefania Catalano

**Affiliations:** 1 Department of Pharmacy, Health and Nutritional Sciences, University of Calabria, Via P. Bucci, Arcavacata Di Rende (CS), 87036 Rende, Cosenza Italy; 2 Centro Sanitario, University of Calabria, Via P. Bucci, Arcavacata Di Rende (CS), 87036 Rende, Cosenza Italy; 3 Departments of Bioinformatics and Pediatrics, Semmelweis University, 1094 Budapest, Hungary; 4 TTK Cancer Biomarker Research Group, 1117 Budapest, Hungary; 5 Department of Clinical Medicine and Surgery, University of Naples Federico II, Naples, Italy; 6 Department of Molecular Medicine and Medical Biotechnology, University of Naples Federico II, Naples, Italy

**Keywords:** Breast cancer, Extracellular vesicles, Obesity, miRNAs, Let-7a

## Abstract

**Background:**

The incidence of obesity, a known risk factor for several metabolic and chronic diseases, including numerous malignancies, has risen sharply in the world. Various clinical studies demonstrate that excessive Body Mass Index (BMI) may worsen the incidence, prognosis, and mortality rates of breast cancer. Thus, understanding the link tying up obesity and breast cancer onset and progression is critically important, as it can impact patients’ survival and quality of life. Recently, circulating extracellular vesicle (EV) derived miRNAs have attracted much attention for their diagnostic, prognostic and therapeutic potential in oncology research. Although the potential role of EV-derived miRNAs in the early detection of breast cancer has been repeatedly mentioned, screening of miRNAs packaged within serum EVs has not yet been reported in patients with obesity.

**Methods:**

Circulating EVs were isolated from normal weight (NW), and overweight/obese (OW/Ob) breast cancer patients and characterized by Transmission Electron Microscopy (TEM), Nanoparticle Tracking Analysis (NTA), and protein marker expression. Evaluation of EV-associated miRNAs was conducted in a screening (RNA-seq) and a validation (qRT-PCR) cohort. Bioinformatic analysis was performed to uncover significantly enriched biological processes, molecular functions and pathways. ROC and Kaplain-Meier survival analyses were used for clinical significance.

**Results:**

Comparison of serum EV-derived miRNAs from NW and OW/Ob patients detected seven differentially expressed miRNAs (let-7a-5p, miR-122-5p, miR-30d-5p, miR-126-3p, miR-27b-3p, miR-4772-3p, and miR-10a-5p) in the screening cohort. GO analysis revealed the enrichment of protein phosphorylation, intracellular signal transduction, signal transduction, and vesicle-mediated transport among the top biological processes. In addition, the target genes were significantly enriched in pathways related to PI3K/Akt, growth hormones, and insulin signalings, which are all involved in obesity-related diseases and/or breast cancer progression. In the validation cohort, qRT-PCR confirmed a significant down-regulation of EV-derived let-7a in the serum of OW/Ob breast cancer patients compared to NW patients. Let-7a levels also exhibited a negative correlation with BMI values. Importantly, decreased let-7a miRNA expression was associated with higher tumor grade and poor survival in patients with breast cancer.

**Conclusion:**

These results suggest that serum-EV derived miRNAs may reflect a differential profile in relation to a patient’s BMI, which, once validated in larger cohorts of patients, could provide insights into novel specific biomarkers and innovative targets to prevent the progression of obesity-mediated breast cancer.

**Supplementary Information:**

The online version contains supplementary material available at 10.1186/s12967-023-04075-w.

## Introduction

The escalating global epidemic of overweight and obesity—‘globesity’—is becoming one of the leading public health crises and has posed serious threats to human health and life quality [1]. Several epidemiologic studies have identified excessive adiposity as a major risk factor for an expanding set of chronic diseases, including different types of malignancies [2]. Excess body weight has been implicated in 15–20% of total cancer-related death [3, 4]. Among overweight/obesity-associated cancers in women, breast cancer represents the most frequently diagnosed cancer and the leading cause of mortality in the world. Indeed, there is expanding evidence showing that high body mass index (BMI) strongly influences risk, prognosis and progression of breast carcinoma and has profound implications on therapeutic management of patients [5–7]. At diagnosis, patients with obesity are more likely to have a worse prognosis [8, 9] and women with a high BMI, regardless of menopausal or hormone receptor status, are at increased risk of breast cancer recurrence [10–13]. In particular, meta-analysis studies found an estimated 35–40% increased risk of disease recurrence or death in obese breast cancer patients compared with normal-weight women [14–16]. Other reports showed that obesity is associated with larger tumor size, positive lymph node status, metastasis development, shorter distant disease-free interval and overall survival [17–21], emphasizing an increased aggressiveness of breast malignancy in patients with high body adiposity. Moreover, a lower benefit of anti-tumor adjuvant therapies (i.e. radiotherapy, chemotherapy, and/or endocrine therapy) has also been encountered in obese women [5, 7]. The mechanisms behind the interplay between obesity and breast cancer progression are likely to be multifactorial and may involve imbalanced adipokine production, abnormal growth factor signaling, a chronic low-grade inflammation, oxidative stress, increased hormone biosynthesis, and the release of several metabolic substrates [7, 22]. Aside from these mediators, extracellular vesicles (EVs) are emerging as new players in this scenario [23–29].

EVs are nanoscale lipid-bilayer enclosed vesicles released from a variety of tissues and cells into biological fluids that are now recognized as a novel axis of intercellular communication, contributing not only to the maintenance of system and organ homeostasis but also to the pathogenesis of diseases. In cancer, EVs interact with, and deliver their contents to target cells in a functional capacity, playing a relevant role in tumor initiation, growth, metastatic dissemination and drug resistance [30–34]. Among others, one of the major exosomal cargo is non-coding RNA, which mainly encompasses miRNAs, short (19–25 nucleotides) RNA molecules that primarily bind to the 3′ untranslated region (UTR) of messenger RNAs to downregulate target proteins [35, 36]. Being the most potent gene expression regulators at the epigenetic, transcriptional, and post-transcriptional level, miRNAs have been found to affect one or several of the cancer hallmarks in a variety of human malignancies, including breast cancer [37–39]. Importantly, compared to free miRNAs in whole blood or serum, miRNAs packaged within EVs remain more stable and reliable since the phospholipid bilayer surrounding EVs can protect them from degradation by nucleases in the body fluids. Thus, EV-miRNAs have attracted much attention for their diagnostic, prognostic and therapeutic utility over the past decade [37, 40]. A pioneer study published in 2016 demonstrated that miR-21 and miR-1246 are selectively enriched in human breast cancer exosomes and their evaluation may be useful for breast cancer diagnosis [41]. Another manuscript showed that EV-miR-21 and EV-miR-105 expression levels were significantly higher in metastatic breast cancer patients versus non-metastatic or healthy controls and EV-miR-222 may discriminate the basal-like and luminal B subtypes from luminal A one [42]; while Sueta et al. revealed 11 differentially expressed EV-miRNAs between recurrent and non-recurrent patients, and four of them (miR-17-5p, miR-93-5p, miR-130a-3p, and miR-340-5p) were significantly associated with recurrence in a logistic regression analysis [43]. More recently, EV-associated miRNAs were identified as predictors of pathological complete response (pCR) in breast cancer patients [44, 45]. On the other hand, the miRNA cargo of EVs is also modified by obesity [46–48], suggesting their role in the development and progression of obesity-related disease. However, in spite of these observations, the potential involvement of circulating EV-derived miRNAs in obesity-associated breast cancers has not been yet investigated.

In the present study, for the first time, we aimed to compare the circulating EV-miRNA expression signatures in normal weight and overweight/obese breast cancer patients and identify potential obesity-related miRNAs that may be associated with progression. This new knowledge may hold great promise for future clinical management of women affected by breast cancer and obesity.

## Materials and methods

### Breast cancer patient population and plasma collection

A total of 45 female patients who were diagnosed with primary breast cancer (BC), between 2016 and 2020, at the Unit of Oncology, Department of Clinical Medicine and Surgery of the University of Naples Federico II, Naples, Italy, were enrolled in this study. Patients with different body mass index (BMI: weight in kilograms divided by height in meters squared, Kg/m^2^) were categorized as it follows: normal weight (NW; 18 > BMI < 24.9 kg/m^2^) and overweight/obese (OW/Ob BMI > 25 kg/m^2^). The study protocol was performed in accordance with the ethical guidelines of the Helsinki Declaration and approved by the Ethical Committee of University of Naples Federico II (n. 107/05). Written informed consent was obtained from every female. Data for the following clinical variables: age, BMI, histology, grading, pre/post-menopausal status, Estrogen Receptor (ER), Progesterone Receptor (PR) and Human Epidermal growth factor Receptor 2 (HER2) status, and Ki67 levels as proliferative index were recorded. Venous blood samples were drawn and serum was obtained after centrifugation, at 3000 rpm for 10 min, and stored in sterile tubes at −80 °C until further processing.

### Extracellular vesicles isolation

**Fig. 1:**
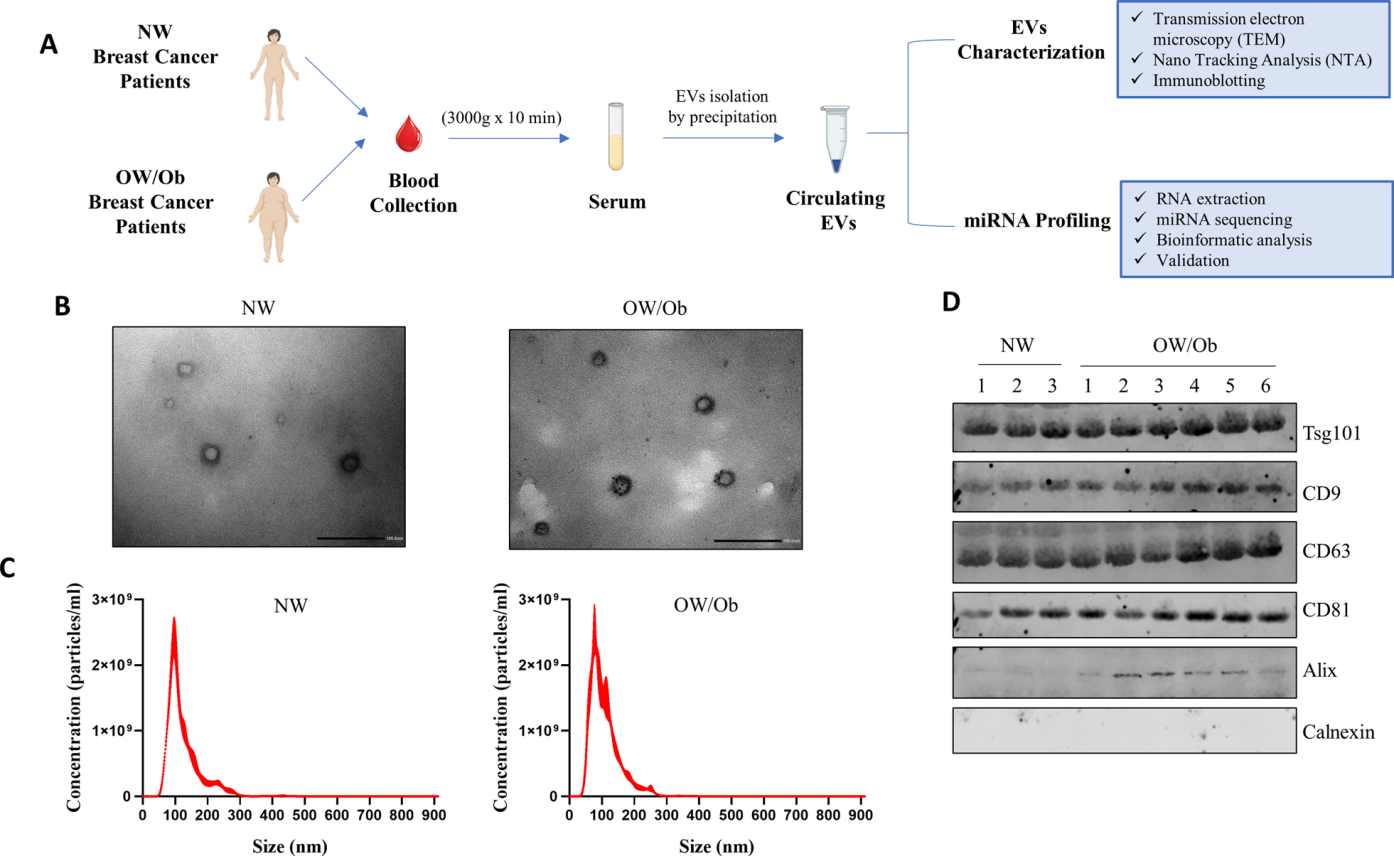
Characterization of extracellular vesicles (EVs) isolated from the serum samples of breast cancer patients with different BMI. **A**. Schematic illustration of the study design. EVs were purified from serum samples of Normal weight (NW; BMI < 25 kg/m^2^), and Overweight/Obese (OW/Ob; BMI ≥ 25 kg/m^2^) breast cancer patients by ExoQuick precipitation system. **B**. Representative images of transmission electron microscopy (TEM) showing EVs isolated from serum of NW and OW/Ob patients. Scale bar, 100 nm. **C**. Representative size distribution profiles and concentration of serum EVs measured by nanoparticle tracking analysis (NTA). **D**. Immunoblot analysis showing expression of the EV hallmarks Tsg101, CD9, CD63, CD81 and Alix in equal amount of EV lysates. The specificity of EV isolation was confirmed using and endoplasmic reticulum marker Calnexin

Extracellular vesicles (EVs) were isolated from serum sample using the ExoQuick isolation agent (System Bioscience, Palo Alto, CA, USA), according to the manufacturer’s instructions. Briefly, serum samples (250 µl) were mixed with ExoQuick reagent and incubated for 30 min at 4 °C to precipitate vesicles. After incubation the samples were centrifuged at 1500 × g for 30 min, the supernatant was discarded, and the pellet was resuspended in sterile phosphate-buffered saline (PBS, 200 μL). The EV samples of the screening cohort have been mixed in equal amounts (3 samples in each pool) to obtain pooled EVs for each group of patients categorized according to the BMI: NW (n = 3 pools) and OW/Ob (n = 6 pools), for a total of 9 NW and 18 OW/Ob patients. The utilization of pooled samples to represent groups of breast cancer patients allowed to obtain enough RNA to yield reproducible reads for miRNA profiling. Figure 1A summarizes the process. Samples were stored at −80 °C until further analysis.

### Transmission Electron Microscopy (TEM)

For TEM analysis, serum EVs were suspended in PBS prior to fixing in 3% glutaraldehyde and transferred to the formovar-coated grids for 20 min, followed by 2% uranyl treatment for 1 min. The grids were examined in a Jeol JEM 1400 Plus electron microscope at 80 kV.

### Nanoparticle Tracking Analysis (NTA)

NanoSight NS300 technology (Malvern Panalytical Ltd., Malvern, UK) was used to analyze the size distribution and the concentration of isolated EVs. The assays were performed according to the recommendation of the instrument’s manufacturer as previously described [49]. Briefly, diluted EV preparation in PBS was passed through the sample chamber and analyzed using a 488 nm laser. For each sample, 5 movies of 60 s were captured and analyzed with Nanosight particle tracking software to calculate particle concentration (particles/ml) and size distribution.

### Immunoblot analysis

EV proteins were extracted adding RIPA lysis buffer (50 mM Tris–HCl, 150 mM NaCl, 1% Nonidet P-40, 0.5% sodium deoxycholate, 2mMsodium fluoride, 2mMEDTA, and 0.1% SDS) containing a mixture of protease inhibitors (aprotinin, phenylmethylsulfonyl fluoride, and sodium orthovanadate) to the isolated EV pellets. Protein concentration was quantified by BCA Protein Assay Kit (Thermo Scientific, UK), according with manufacturer’s instructions. Equal amounts of EV extracts were resolved on 11% SDS–polyacrylamide gel as previously described [50], and probed with anti-Tsg101 (sc-7964, Santa Cruz Biotechnology, Dallas, TX, USA), anti-CD9 (ab236630, Abcam, Cambridge, UK), anti-CD63 (ab213092, Abcam), anti-CD81 (ab155760, Abcam), anti-Alix (ab186429, Abcam) and anti-Calnexin (sc-11397, Santa Cruz Biotechnology) overnight at 4 °C, followed by secondary IRDye 800CW goat anti-mouse or anti-rabbit antibodies (LI-COR, Germany). The images were acquired using Odissey FC (LI-COR, Lincoln, NE, USA).

### RNA isolation

Total RNA was extracted from purified vesicles using the miRNeasy Mini Kit (Qiagen, Hilden, Germany), following the manufacturer’s instructions. Purified RNA was eluted in RNase-free water and stored at −80 °C until use. RNA concentration was determined by using Qubit Fluorometer (Thermo Fisher Scientific, Waltham, MA, USA) and its quality was assessed with the TapeStation 4200 (Agilent Technologies, Santa Clara, CA, USA).

### Profiling of miRNAs in EVs

The miRNA profile of circulating EVs in breast cancer patients was performed by RNA-seq. Indexed libraries were prepared with NEXTFLEX Small RNA-Seq Kit v3 (PerkinElmer, Waltham, MA, USA) according to the manufacturer’s instructions. Libraries were quantified using the TapeStation 4200 (Agilent Technologies) and Qubit Fluorometer (Thermo Fisher Scientific), then pooled such that each index-tagged sample was present in equimolar amounts. The pooled samples were subject to cluster generation and sequencing using an Illumina NextSeq 550 Dx System (Illumina, San Diego, CA, USA) in a 1 × 75 single-end format. The raw sequencing files are deposited in the NCBI’s Gene Expression Omnibus and are available through GEO Series accession number GSE222681.

### Identification of miRNA target genes and their molecular pathways

The putative miRNA target genes were identified for each miRNA separately using mirDB (https://doi.org/10.1093/nar/gkz757), with default parameters. The complete list of all miRNA target genes was termed as the “entire target signature”. For each setting, DAVID functional annotation was performed to uncover significantly overrepresented biological processes and molecular functions (https://doi.org/10.1038/nprot.2008.211). GO terms with a false discovery rate (FDR) values of < 0.05 and a p < 0.05 were considered.

### Construction of the regulatory network

Search Tool for the Retrieval of Interacting Genes/Proteins (STRING, https://string-db.org/) database was used for online retrieval of known protein–protein interactions (PPI) [51]. We submitted the entire list of putative target genes to STRING and set the parameters including a minimum required interaction score > 0.9 (highest confidence).

### qRT-PCR

Quantitative RT-PCR (qRT-PCR) was used to validate the results of the bioinformatics analysis. Complementary DNA was synthesized with the TaqMan^™^ Advanced miRNA cDNA Synthesis Kit (Applied Biosystems, Thermo Fisher Scientific) and miRNA expression levels were analysed using TaqMan^™^ MicroRNA assay probes (Additional file 1: Table S1) and TaqMan^™^ Universal PCR Master Mix (Applied Biosystems) according to the manufacturer’s instructions. cDNA samples were diluted 1:10 and distributed in triplicates of 5 µl each in the plate. A cDNA negative control was included in each assay. RT-q-PCR was performed on a QuantStudio™ 3 (Thermo Fisher Scientific) with the following protocol: 95 °C for 20 s, 40 cycles of 95 °C for 1 s, and 60 °C for 20 s. The threshold value adopted was 0.2. Normalization was performed using the spike-in cel-miR-39 or the endogenous miR-191 or miR-484, frequently used by others [52–54]. The relative expression of miRNA expression was calculated using the comparative cycle threshold 2ΔΔ^−Ct^ method.

### Statistical analysis

For analysis of miRNA profiling, the bioinformatic tool cutadapt (version 2.5) was used to remove the adapter sequence according to the kit used. The tool sRNAbench was used to remove the low-quality reads and to obtain the miRNA expression profiling respect to miRBase database (version22—GRCh38—GCA_000001405.15). Reads that had a sum read count less than ten were excluded, in all samples. To identify differentially expressed miRNAs, the DESeq2 algorithm was used. miRNAs with p ≤ 0.05, and |Fold-Change| (|FC|) ≥ 1.5, were considered as differentially expressed between the two conditions. Linear regression was analyzed to evaluate the relationship between hsa-let-7a levels and BMI. The regression coefficient was used to graph the straight line that most closely described the association between variables, and the statistical correlation was evaluated by Pearson’s correlation. Receiver operating curves (ROC) were constructed and area under the curve (AUC) with 95% confidence interval, sensitivity and specificity were calculated to evaluate low hsa-let-7a levels as predictor of tumor grade (G3) or Ki67 positivity (Criterion: ≥ 20%). Data were analyzed for statistical significance (p < 0.05) using a two-tailed student’s Test, performed by GraphPad-Prism 7 software program (GraphPad Inc., San Diego, CA, USA).

### Survival Kaplan–Meier analysis

Kaplan–Meier plots were generated using the Kaplan–Meier plotter online tool by using patient datasets as described [55]*.*

## Results and discussion

### Isolation and characterization of extracellular vesicles derived from breast cancer patients with different BMI

Circulating extracellular vesicles (EVs) were isolated from two different screening cohorts: breast cancer patients with normal weight (indicated as NW patients), and overweight/obese patients (indicated as OW/Ob patients). The workflow of our study is illustrated in Fig. 1A. The participants’ clinical information of age, BMI, menopausal status, tumor characteristics, and classification are summarized in Table 1.

**Table 1:** Clinicopathological characteristics of breast cancer patients with different BMI included in the screening cohort

Clinical variables	*Screening cohort* (n)	
	NW (9) (BMI < 24.9 kg/m^2^)	OW/Ob (18) (BMI ≥ 25 kg/m^2^)
*Age, y*		
Median	44	55
Range	30–73	36–76
BMI Kg/m^2^, mean ± SD	21.7 ± 2.5	30.6 ± 4.06
*Histology, %*		
In situ ductal carcinoma	0	5.5
Invasive ductal carcinoma	100	83.3
Invasive lobular carcinoma	0	11.2
*Grading, %*		
Well differentiated (G1)	0	11.1
Moderately differentiated (G2)	33.3	55.6
Poor/undifferentiated (G3)	44.4	22.2
Unknown	22.2	11.1
*Menopausal status, %*		
Premenopausal	88.8	50
Postmenopausal	11.2	50
*Estrogen receptor status, %*		
Negative	22.2	5.5
Positive (> 1%)	77.7	94.4
*Progesterone receptor status, %*		
Negative	22.2	22.2
Positive (> 1%)	77.7	77.7
*HER2/Neu status, %*		
Negative	77.7	72.2
Positive	22.2	27.7
*Ki67, %*		
< 14	0	11.2
≥ 14	100	88.8

*NW* Normal weight, *OW/Ob* Overweight/Obese, *BMI* Body Mass Index, *SD* Standard Deviation, *HER2/neu* Human Epidermal Growth Factor 2

The EV enriched fractions were successfully extracted from the serum of all patients, and characterized by their shapes, size and protein markers (Fig. 1). TEM and NTA analysis showed that EVs in the isolated fractions were oval or bowl-shaped with a size range between 94 and 130 nm (Fig. 1B, C, respectively). In accordance with the Minimal Information for Studies of Extracellular Vesicles (MISEV) 2018 [56], enrichment of the EV markers Tsg101, CD9, CD63, CD81, and Alix were all detected in the EV enriched fractions derived from the serum (Fig. 1D). On the contrary, Calnexin, a negative marker for EVs, was absent in our isolated EVs (Fig. 1D). Besides, the protein of the EV-enriched fraction samples was also evaluated. An EV-associated protein concentration of 209.5 ± 25.7 µg per mL serum was reported.

### miRNA expression in extracellular vesicles derived from breast cancer patients with different BMI

**Fig. 2:**
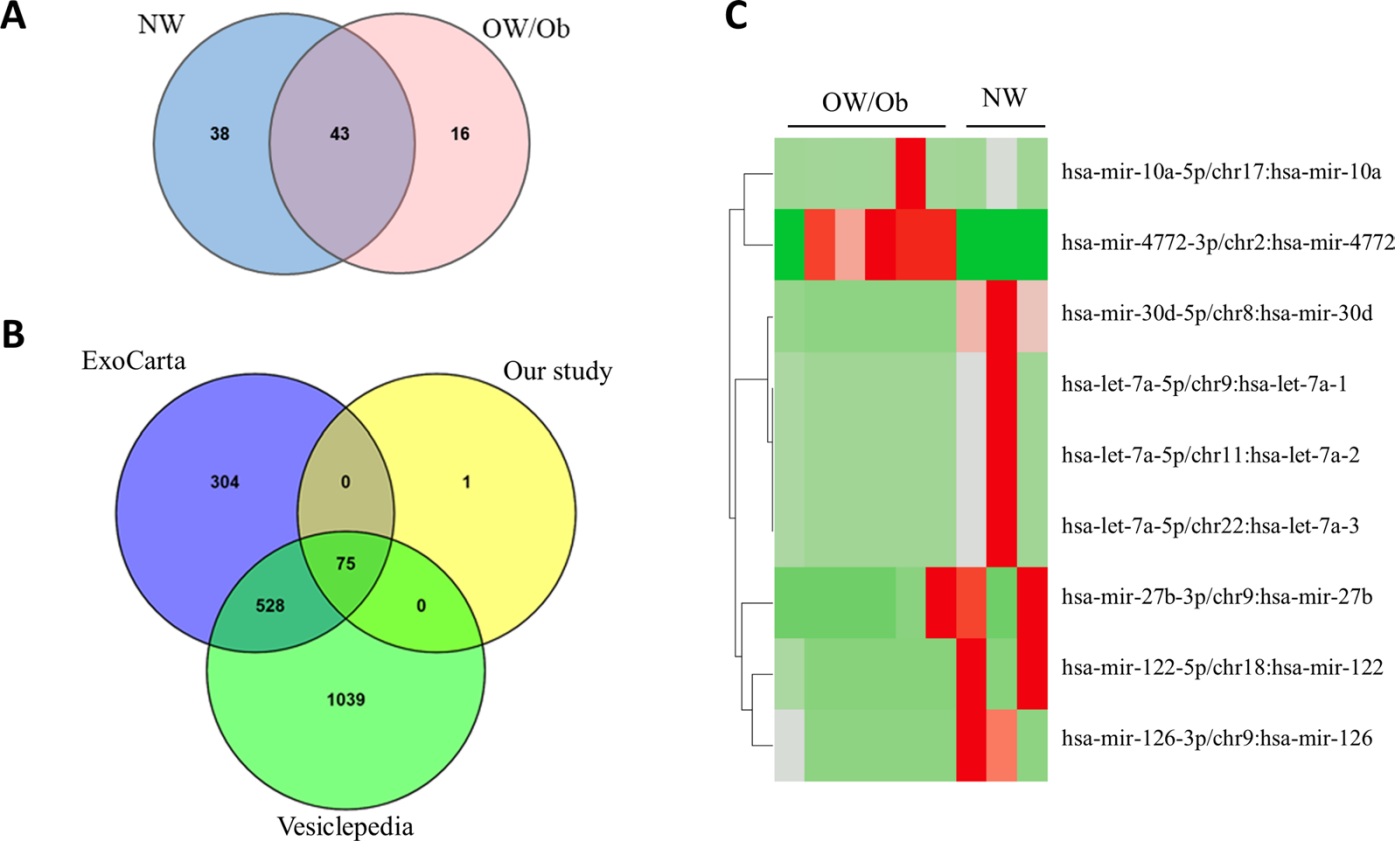
Differentially expressed EV-miRNAs in breast cancer patients with different BMI assessed by RNA-seq. **A**. Venn diagram of identified EV-miRNAs in serum of NW and OW/Ob breast cancer patients. **B**. Venn diagram of the known miRNAs among our study, vesicular Vesiclepedia and ExoCarta databases. **C**. Heatmap of the differentially expressed EV-miRNAs in NW and OW/Ob breast cancer patients. The red and the green color scale indicate an increase and a decrease in miRNA expression levels, respectively

To determine the global expression profile of the EV-miRNAs isolated from the serum of our cohort, RNA was extracted from EVs and miRNA sequencing was performed. Raw data of small RNA sequencing were filtered and normalized. For each sample, no less than 10 clean reads were used. A total of 97 different miRNA probes were identified among all samples (Fig. 2A). Almost all of the known miRNAs were already included in Vesiclepedia [57] and ExoCarta [58] vesicular databases as related to EVs or exosomes from human samples (Fig. 2B). The hsa-let-7 family appeared as one of the most representative miRNA groups among our data set (~ 13%). The whole list of all identified miRNAs has been submitted to the Vesiclepedia database and entitled as this manuscript.

Statistical analyses of the miRNAs identified by sequencing showed seven differentially expressed miRNAs in the screening cohort. In particular, four miRNAs were down-regulated (let-7a-5p, miR-122-5p, miR-30d-5p, and miR-126-3p), and three miRNAs were up-regulated (miR-27b-3p, miR-4772-3p, and miR-10a-5p) in OW/Ob population compared to NW patients (Table 2). A heatmap displaying results of the supervised hierarchical clustering is shown in Fig. 2C. These seven miRNAs were selected for further analysis.

**Table 2:** List of differentially-expressed mature miRNAs in circulating extracellular vesicles of Overweight/Obese breast cancer patients compared to patients with Normal Weight

miRNAs	Log2 Fold Change	*adjusted p value*
hsa-let-7a-5p	−17.52	1.19E-02
hsa-miR-122-5p	−17.19	1.19E-02
hsa-miR-30d-5p	−18.89	1.19E-02
hsa-miR-126-3p	−16.08	1.89E-02
hsa-miR-27b-3p	14.64	1.19E-02
hsa-miR-4772-3p	10.20	1.89E-02
hsa-miR-10a-5p	9.97	2.31E-02

Log2 fold change, fold change in logarithmic scale

### Potential function of EV-derived miRNAs identified in the screening cohort

We first carried out a bibliographic search of those seven significantly and differentially expressed miRNAs to examine any correlations with breast cancer progression and obesity Table 3, [52, 59–86].

**Table 3:** Summary of the literature for the differentially expressed EV-miRNAs between NW and OW/Ob breast cancer patients

miRNAs	Previously reported in the literature	References
hsa-let-7a-5p	Decreased expression of circulating EV-derived miRNA-let-7a in inflammatory breast cancer (IBC) patients compared to non-IBC and health controls	[52]
	Decreased expression of circulating miRNA-let-a in breast cancer patients compared to health controls	[59]
	Decreased expression of circulating miRNA-let-a in post-operative breast cancer patients compared to control subjects	[60]
	Decreased expression of circulating miRNA-let-a in breast cancer compared to the control group and the benign breast lesions group. Negative correlation of circulating miRNA-let-a with development of metastases	[61]
	Decreased expression of circulating miRNA-let-a in non-metastatic breast cancer patients following the common treatments, such as surgery, chemotherapy, and radiotherapy compared to the control ones. Increased expression of circulating miRNA-let-a in non-metastatic breast cancer patients after surgery, chemotherapy, and radiotherapy treatments than the pre-treatment	[62]
	Decreased expression of circulating miRNA-let-a in diabetic patients compared to healthy individuals	[63]
	Down-regulation of peripheral blood mononuclear cells (PBMC)-derived miRNA let-7a in children with metabolic-associated fatty liver disease (MAFLD) and insulin resistance	[64]
	Decreased expression of circulating miRNA-let-a in obese subjects with metabolic syndrome (MetS) compared to non-Met patients	[65]
hsa-miR-122-5p	Up-regulation of plasma‐derived exosomes miRNA‐122 in breast cancer patients compared to normal controls	[66]
	Positive correlation of circulating miR-122 with clinical outcomes including neoadjuvant chemotherapy, response and relapse with metastatic disease	[67]
	Increased expression of circulating miRNA-122 in locally advanced primary breast cancer patients treated with neoadjuvant chemotherapy	[68]
	Positive correlation of increased expression of circulating miRNA-122 with obesity, insulin resistance, metabolic syndrome, type 2 diabetes, adverse lipid profile and non-alcoholic fatty liver disease	[69, 82–84]
	Negative correlation of circulating miRNA-122 levels with brown adipose tissue activity and serum HDL-cholesterol; positive correlation with BMI, body fat mass, total cholesterol and triglyceride serum levels	[70]
	Increased expression of circulating miRNA-122 in subjects with the metabolic syndrome (MetS) compared to non-Met ones; decreased expression of circulating miRNA-122 in response to weight loss	[71]
	Increased expression of circulating miRNA-122 in children with obesity compared to health controls	[72, 85]
	Positive correlation of circulating miRNA-122 levels with clinical and biochemical markers of obesity and insulin resistance in adolescents	[73]
	Increased expression of circulating miRNA-122 in children with nonalcoholic fatty liver disease compared to healthy controls	[74]
	Decreased expression of circulating miRNA-122 in women with pregestational obesity and gestational obesity compared to normal pregnancies (control)	[75]
hsa-miR-30d	Positive correlation of circulating miRNA-30d levels with clinical and biochemical markers of obesity and insulin resistance in adolescents	[73]
hsa-miR-126-3p	Decreased expression of circulating miRNA-126 in type 2 diabetes	[76, 86]
	Increased expression of circulating miRNA-126 in subjects with obesity	[77]
hsa-miR27b-3p	Decreased expression of circulating EV-derived miRNA- 27b in triple negative breast cancer patients compared to Her2-positive	[78]
	Positive correlation of circulating miRNA-27b levels with body mass index in children	[72]
	Increased expression of circulating miRNA-27b in obese children compared to healthy controls	[79]
hsa-miR-4772-3p	Positive correlation of peripheral blood mononuclear cells (PBMC)-derived miRNA-4772 levels with the magnitude of weight loss in subjects with obesity	[80]
hsa-miR10a-5p	Increased expression of circulating miRNA-10a in women with clinically stable metastatic breast cancer responders to post lifestyle intervention (diet and physical activity) compared with their baseline and non-responders	[81]

To date, no studies investigating the role of EV-derived miRNAs in obesity-related breast cancer were previously described in the literature and few studies have these selected miRNAs, mainly circulating ones, independently associated to either breast cancer or obesity. In particular, only let-7a, miRNA 27b, miR-122-5p, and miR-126-3p were reported to exist in breast cancer patients’ EVs and function in this context. Specifically, EV-derived let-7a, and miRNA 27b were found to be downregulated in inflammatory breast cancer (IBC) patients compared to non-IBC and healthy controls [52] and in triple-negative breast cancer patients compared to HER2-positive ones [78]. Exosome-derived miR-122-5p was up-regulated in breast cancer women compared to normal controls [66], while expression levels of exosomal miR-126 did not change between breast cancer and control individuals [87]. Other reports demonstrated different expression of our identified circulating miRNAs in breast cancer patients’ plasma or serum and their correlation with clinical outcomes [59–62, 67, 68, 78, 81, 87–90]. For instance, expression levels of serum miRNA let-7 were significantly decreased in patients with breast cancer compared to control and to benign breast lesion groups, and negatively correlated with development of metastases [61]. Interestingly, some of the selected circulating miRNAs, especially circulating let-7a, miR-122, miR-126, and miR-27b, were also associated with clinical and biochemical markers of obesity, insulin resistance, metabolic syndrome, type 2 diabetes, and lipid profile [63–65, 69–77, 79, 82–84, 91, 92].

**Fig. 3:**
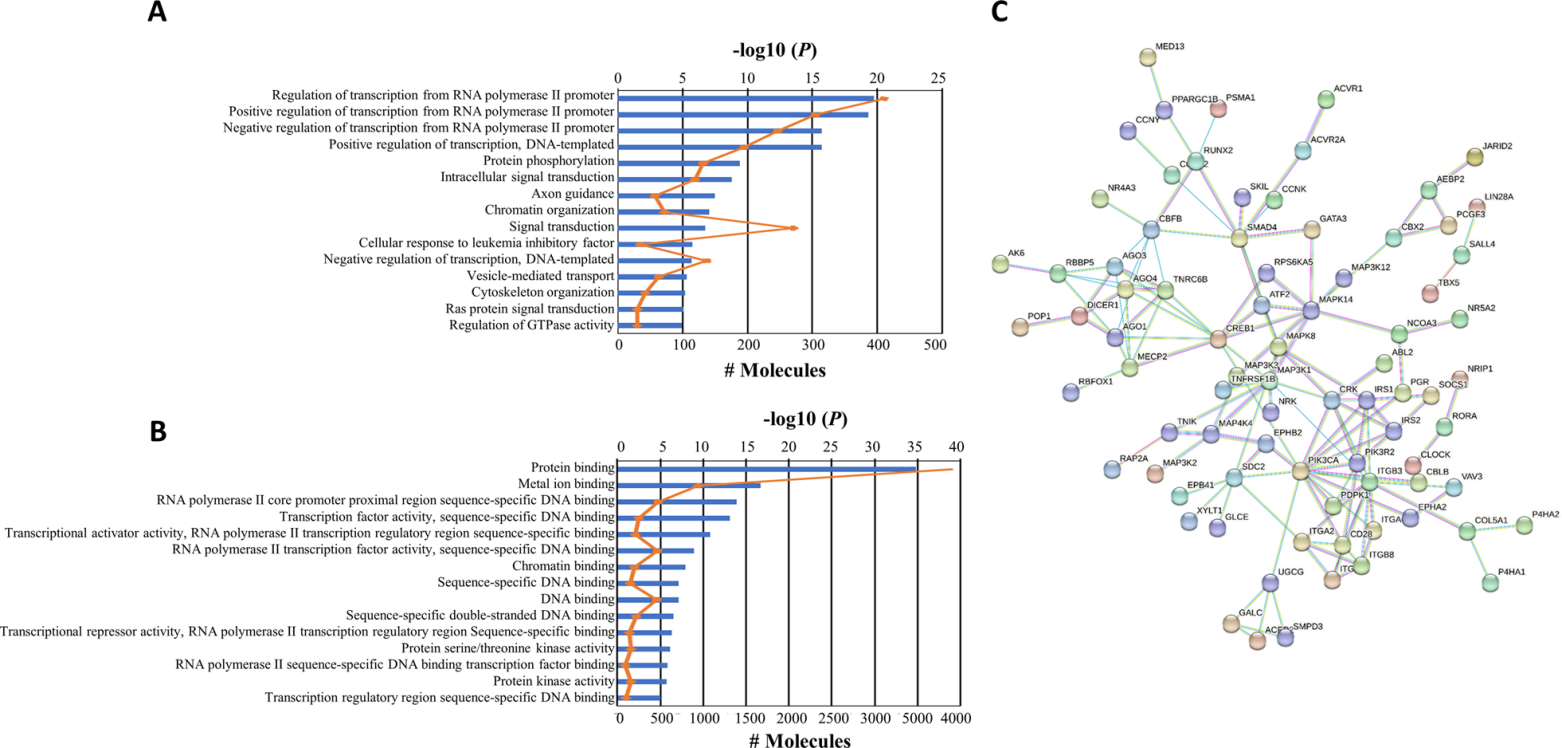
Gene Ontology (GO) analysis of the differentially expressed EV-miRNA target genes. GO enrichment of biological processes (**A**) and molecular functions (**B**) of EV-miRNA target genes in OW/OB vs NW breast cancer patients. DAVID database was used for GO-term functional annotation analysis. Data showed the top 15 significant GO terms with FDR (false discovery rate) values of < 0.05 and a p-value of < 0.05. **C**. Protein–protein interaction (PPI) analysis of the deregulated EV-miRNA target genes constructed using the Search Tool for the Retrieval of Interacting Gene (STRING; https://string-db.org/) database. Each node indicates a protein module; the edges represent protein interactions. Different color indicates different type of interactions. (Cyan-from curated databases; Pink-experimentally determined; Khaki- text mining). PPIs with a p < 0.05 were considered statistically significant

To define their potential functions, these seven significantly and differentially expressed EV-derived miRNAs were then screened for miRNA target genes’ prediction using mirDB software. The entire signature comprises 2754 genes in the down-regulated cohort and 2176 genes in the up-regulated one. These genes were analysed and networked by DAVID functional annotation to uncover significantly overrepresented biological processes and molecular functions (Fig. 3A, B, respectively). As expected, most of the analysed genes were related to the regulation of transcription (p = 1.80E-20). GO analysis for biological processes also revealed the enrichment of protein phosphorylation (p = 3.68E-10), intracellular signal transduction (p = 1.54E-09), and signal transduction (p = 1.88E-7). Moreover, the biological process indicated as vesicle-mediated transport (p = 4.77E-06) was also enriched. Among the molecular functions, protein binding was the most represented one (p = 1.40E-35). To understand the regulatory network of protein targets of EV-miRNAs found deregulated in our OW/Ob breast cancer patient population, we performed protein–protein interaction (PPI) analysis via the STRING database. The entire list of putative target genes was input into STRING database and achieved a PPI network of 426 nodes and 150 edges, with PPI enrichment p-value < 1.29 × 10^–10^ (Fig. 3C). Moreover, using the value of node degree > 5 as filtering criteria, we identify different (eighteen) hub genes (*AGO1, AGO3, AGO4, CD28, MAPK8, MECP2, PIK3R2, CBFB, IRS1, SDC2, TNRC6B, CRK, MAP3K1, MAPK14, SMAD4, CREB1, ITGB3* and *PIK3CA*) among which PIK3CA showed the highest degree nodes (# 14). In addition, the target genes were significantly enriched in pathways mediating PI3K/Akt, growth hormones, and insulin signaling (Additional file 1: Table S2), that all have been closely correlated with obesity-related diseases and/or breast cancer progression [93–96].

### Comparison of the EV-derived miRNA signatures in the validation cohort

To validate the results obtained in the screening cohort, we extended our investigation to a second series of breast cancer patients having different BMI values (validation cohort, Table 4).

**Table 4:** Clinicopathological characteristics of breast cancer patients of the validation cohort

Clinical variables	*Validation cohort* (n)	
	NW (6) (BMI < 24.9 kg/m^2^)	OW/Ob (12) (BMI ≥ 25 kg/m^2^)
*Age, y*		
Median	50	64
Range	34–73	46–83
BMI Kg/m^2^, mean ± SD	23 ± 1.8	30.6 ± 3
*Histology, %*		
In situ ductal carcinoma	0	0
Invasive ductal carcinoma	83	92
Invasive lobular carcinoma	0	8
*Grading, %*		
Well differentiated (G1)	17	0.8
Moderately differentiated (G2)	33	25
Poor/undifferentiated (G3)	33	58
Unknown	17	8.3
*Menopausal status, %*		
Premenopausal	50	17
Postmenopausal	50	83
*Estrogen receptor status, %*		
Negative	17	25
Positive (> 1%)	83	75
*Progesterone receptor status, %*		
Negative	33	33
Positive (> 1%)	67	67
*HER2/Neu status, %*		
Negative	50	92
Positive	33	8.3
*Ki67, %*		
< 14	33	8.3
≥ 14	67	92

*NW* Normal weight, *OW/Ob* Overweight/Obese, *BMI* Body Mass Index, *SD* Standard Deviation, *HER2/neu* Human Epidermal Growth Factor 2

**Fig. 4:**
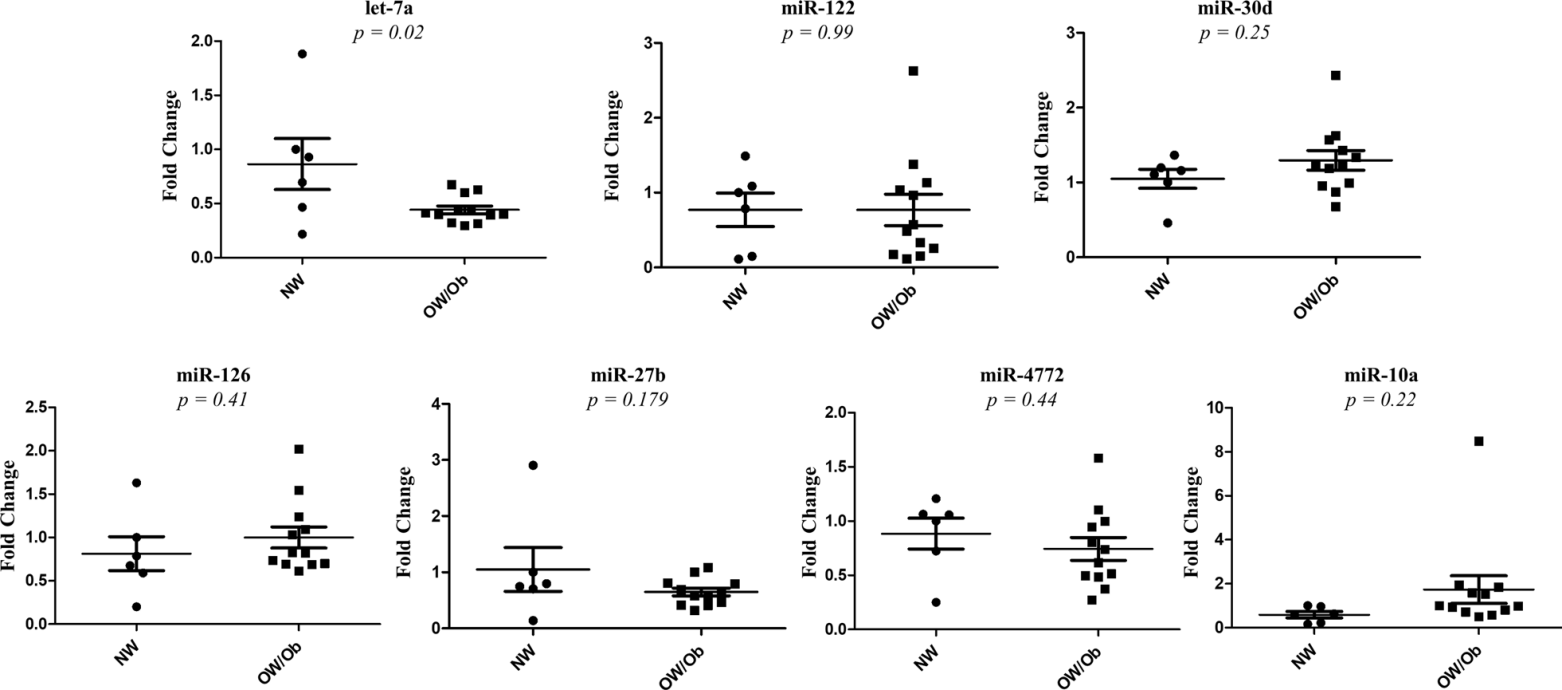
Quantitative RT-PCR of miRNA expression profiling data in validation cohort. qRT-PCR was conducted to measure the mRNA expression levels of let-7a, miR-122, miR-30d, miR-126, miR-27b, miR-4772 and miR-10a. The mean threshold cycle (Ct) was determined based on triplicate reactions. The 2^−ΔΔCt^ method was used to calculate the fold differences in EV-miRNA expression among the tested samples. miRNA expression was normalized against the spike-in cel-miR-39 as well as the endogenous miR-484 and miR-191. Data were reported as mean ± SEM. Significance was calculated with GraphPad Prism software by using the two-tailed t-test

Again, EVs were extracted from the serum of these patients, and characterized by TEM, NTA and immunoblotting as previously described. Realtime PCR analysis confirmed a significant down-regulation of EV-derived let-7a in OW/Ob breast cancer patients compared to NW women. In contrast, in the breast cancer validation cohort no significant differences were detected in the levels of miR-122, miR-30d, miR-126, miR-27b, miR-4772, and miR-10a (Fig. 4).

**Fig. 5:**
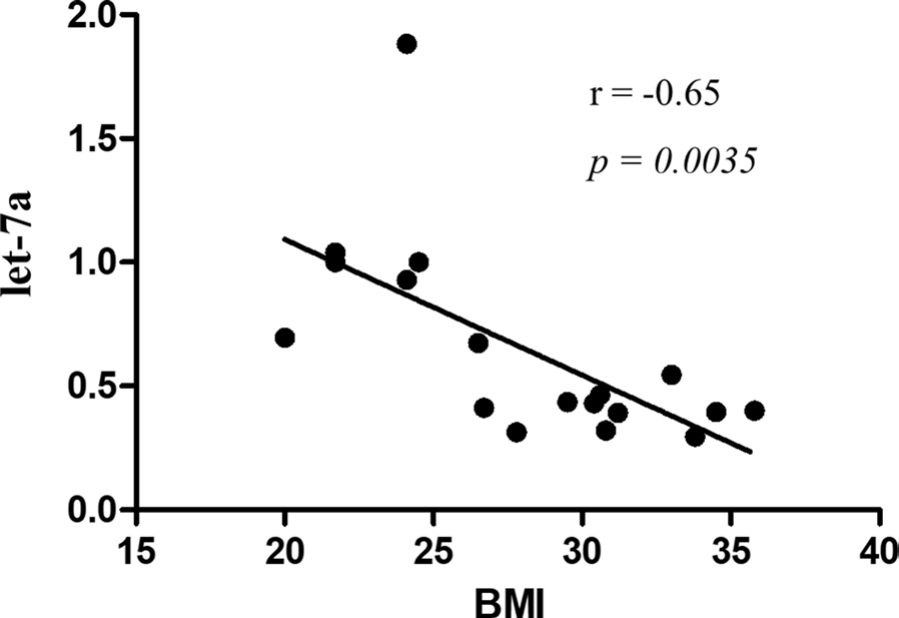
Correlations analysis between let-7a and BMI values in a validation cohort. Let-7a and BMI association was analyzed by Pearson’s correlation test. Linear regression graph, correlation coefficient (r), and statistical significance (p) were reported

To corroborate the association of the expression levels of let-7a from circulating EVs and patients’ BMI, correlation and linear regression analyses were carried out. We found a negative correlation between let-7a levels and BMI values (r = −0.65 and p = 0.0035) in our population (Fig. 5). These results agree with previous literature showing that the levels of circulating miRNAs from let-7 family, mainly let-7a, were lower in obese subjects compared to healthy individuals. Indeed, circulating let-7a was found to be reduced in patients affected by diabetes [63], or metabolic syndrome [65], and in children with metabolic-associated fatty liver disease and insulin resistance [64].

### Association of let-7a with clinical outcome in breast cancer patients

**Fig. 6:**
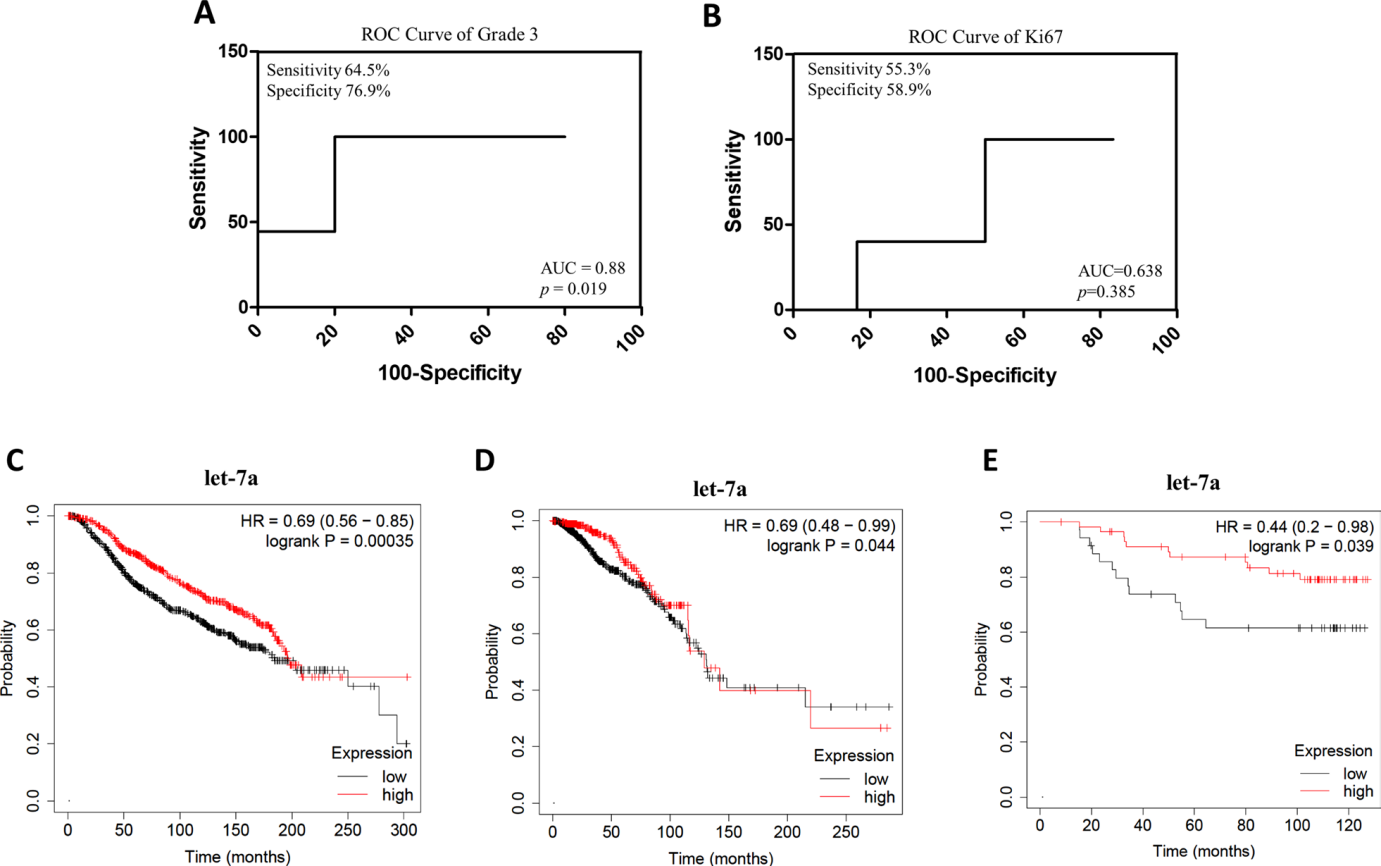
Role of Let-7a in breast cancer patients. Receiver operating characteristic (ROC) curves of let-7a as predictor of tumor grading (G3) (**A**), and Ki67 level (Criterion: ≥ 20%) (**B**). AUC: area under the curve. Kaplan–Meier survival analysis relating let-7a levels and overall survival in breast cancer patients using Metabric (**C**), TGCA (**D**), and GSE (**E**) datasets. *HR* hazard ratio

To verify the potential of let-7a as a suitable classifier in predicting prognostic characteristics, including tumor grading and the proliferation marker Ki67 levels, receiver operating characteristic (ROC) analysis was performed, and the area under curve (AUC) was calculated. ROC analysis demonstrated that let-7a could discriminate G3 vs G1/G2 grade of breast cancer with AUC of 0.88 (p = 0.019), indicating a good performance of this individual miRNA (Fig. 6A). However, let-7a is less efficient in discriminating high Ki67 percentage in breast cancer patients with different BMI (Fig. 6B, AUC = 0.63 and p = 0.38).

Finally, using the Kaplan–Meier plot online platform [55], we investigated whether the let-7a miRNA expression may be associated with breast cancer patient survival. We observed a shorter overall survival of patients with low levels of let-7a, with long-rank p value of 0.00035 in the Metabric dataset (Fig. 6C). The value of miRNA let-7a was subsequently validated in other two independent datasets (TGCA and GSE19783), which confirmed the findings driven from the initial Metabric cohort (Fig. 6D and E, respectively). Since no databases correlating specific EV-miRNA cargos and patients’ survival have been available and considering that EV-enclosed and whole plasma cell-free miRNAs may correlate in some settings [97], it is evident that the value of let-7a in EVs as a potential obesity-related miRNA associated with breast cancer progression deserves further attention.

### Study limitations

Our findings provide a first line of evidence of a difference in circulating miRNA-EV profiling of breast cancer patients according to their BMI. Indeed, there are potential limitations to consider when interpreting the results. First, the relatively small sample size of the populations may hamper definitive conclusions. Moreover, although BMI is a practical and widely chosen indicator for diagnosing obesity and evaluating its negative impact among individuals, the stratification of patients’ subgroups could take into account alternative measures (i.e. waist/hip ratio, or waist circumference) considered as better anthropometric parameters than BMI to predict the risk of obesity-related diseases [98–100]. Certainly, future studies in a larger cohort classified by both BMI and alternative anthropometric characteristics could further clarify the role of EV-let-7a in obesity-related breast cancers.

## Conclusions

EV-miRNA cargoes are emerging as potential diagnostic and prognostic biomarkers that can be easily detected using non-invasive procedures, including liquid biopsies. In this study, we determined the miRNA expression profile of EVs derived from the serum of normal weight breast cancer patients and overweight/obese patients and identified EV-let-7a as a novel and not yet studied miRNA that may be associated with progression of obesity-mediated breast cancer. We found a significant down-regulation of EV-derived let-7a among patients with obesity along with a negative correlation between let-7a levels and BMI values in our population. Importantly, low let-7a miRNA expression may predict increased tumor grade and poor patient survival. Because of obesity and its pathophysiological sequelae on the rise, this new knowledge could provide specific biomarkers and innovative targets that may allow a personalized management of patients affected by breast cancer and increased adiposity.

## Supplementary Information

**Additional file 1: Table S1.** TaqMan Advanced miRNA Assays used for miRNA relative quantification (Thermo Fisher Scientific). The manufacturer assay ID is reported. **Table S2.** Enriched KEGG pathways of the deregulated EV-miRNA target genes.

## Data Availability

All the data are available in a public, open-access repository.

## Abbreviations

NW: Normal weight
OW/Ob: Overweight/Obese
BMI: Body mass index
EV: Extracellular vesicles
SEM: Standard error of the mean
HER2/neu: Human epidermal growth factor 2
AGO1: Argonaute RISC component 1
AGO3: Argonaute RISC catalytic component 3
AGO4: Argonaute RISC component 4
CD28: CD28 molecule
MAPK8: Mitogen-activated protein kinase 8
MECP2: Methyl-CpG binding protein 2
PIK3R2: Phosphoinositide-3-kinase regulatory subunit 2
CBFB: Core-binding factor subunit beta
IRS1: Insulin receptor substrate 1
SDC2: Syndecan 2
TNRC6B: Trinucleotide repeat containing adaptor 6B
CRK: CRK proto-oncogene, adaptor protein
MAP3K1: Mitogen-Activated protein kinase Kinase Kinase 1
MAPK14: Mitogen-Activated Protein Kinase 14
SMAD4: SMAD family member 4
CREB1: CAMP responsive element binding protein 1
ITGB3: Integrin subunit beta 3
PIK3CA: Phosphatidylinositol-4,5-bisphosphate 3-kinase catalytic subunit alpha
PI3K: PhosphatidylInositol 3-Kinase
Akt: Protein-chinasi B
