# Supplementary material for: Analysis of circulating extracellular vesicle derived microRNAs in breast cancer patients with obesity: a potential role for Let-7a
Source: J Transl Med. 2023 Mar 31;21:232. doi: 10.1186/s12967-023-04075-w (PMC10064709; doi:10.1186/s12967-023-04075-w)
Supplement: Supplementary file 1 — Additional file 1: Table S1. TaqMan Advanced miRNA Assays used for miRNA relative quantification (Thermo Fisher Scientific). The manufacturer assay ID is reported. Table S2. Enriched KEGG pathways of the deregulated EV-miRNA target genes. [file 12967_2023_4075_MOESM1_ESM.docx]

**Additional file 1**

**Table S1.** TaqMan Advanced miRNA Assays used for miRNA relative quantification (Thermo Fisher Scientific). The manufacturer assay ID is reported.

| **Assay** | **Assay ID** | **Target sequence** |
| --- | --- | --- |
| hsa-let-7a-5p | 478575_mir | UGAGGUAGUAGGUUGUAUAGUU |
| hsa-miR-10a-5p | 479241_mir | UACCCUGUAGAUCCGAAUUUGUG |
| hsa-miR-27b-3p | 478270_mir | UUCACAGUGGCUAAGUUCUGC |
| hsa-miR-30d-5p | 478606_mir | UGUAAACAUCCCCGACUGGAAG |
| hsa-miR-122-5p | 477855_mir | UGGAGUGUGACAAUGGUGUUUG |
| hsa-miR-126-3p | 477887_mir | UCGUACCGUGAGUAAUAAUGCG |
| hsa-miR-4772-3p | 478121_mir | CCUGCAACUUUGCCUGAUCAGA |
| hsa-miR-484 | 478308_mir | UCAGGCUCAGUCCCCUCCCGAU |
| hsa-miR-191-5p | 477952_mir | CAACGGAAUCCCAAAAGCAGCUG |
| cel-miR-39-3p | 478293_mir | UCACCGGGUGUAAAUCAGCUUG |

| **KEGG ID** | **KEGG Term** | **Count** | **FDR** | **Genes** |
| --- | --- | --- | --- | --- |
| hsa04151 | PI3K-Akt signaling pathway | 17 | 0.0425 | *PIK3R2, ITGB8, PIK3CA, ATF2, CDK6, ITGA2, TSC1, IRS1, PDPK1, EPHA2, PRKAA2, ITGA8, ITGA6, CREB1, ITGB3, PHLPP2, PRLR* |
| hsa04935 | Growth hormone synthesis, secretion and action | 12 | 0.0075 | *PIK3R2, MAPK14, PIK3CA, ATF2, CRK, IRS1, SOCS1, IRS2, PLCB4, MAPK8, MAP3K1, CREB1* |
| hsa04550 | Signaling pathways regulating pluripotency of stem cells | 12 | 0.0111 | *PIK3R2, MAPK14, ACVR2A, ACVR1, PIK3CA, JARID2, SMAD4, HOXA1, PCGF3, ID4, SKIL, FZD3* |
| hsa04910 | Insulin signaling pathway | 12 | 0.0111 | *PIK3R2, PIK3CA, CBLB, PDE3B, TSC1, CRK, IRS1, SOCS1, PDPK1, PRKAA2, IRS2, MAPK8* |
| hsa05206 | MicroRNAs in cancer | 12 | 0.0129 | *PIK3R2, PIK3CA, CDK6, CRK, IRS1, SOCS1, MDM4, IRS2, DICER1, FZD3, ITGB3, RPS6KA5* |
| hsa04810 | Regulation of actin cytoskeleton | 12 | 0.0463 | *PIK3R2, ITGB8, PIK3CA, ITGA2, CFL2, CRK, SSH1, VAV3, PIP4K2A, ITGA8, ITGA6, ITGB3* |
| hsa04068 | FoxO signaling pathway | 11 | 0.0111 | *PIK3R2, MAPK14, PIK3CA, IRS1, HOMER2, SMAD4,PDPK1, PRKAA2, IRS2, MAPK8, FBXO32* |
| hsa04722 | Neurotrophin signaling pathway | 11 | 0.0111 | *PIK3R2, MAPK14, PIK3CA, CRK, IRS1, PDPK1, SH2B3, MAP3K3, MAPK8, MAP3K1, RPS6KA5* |
| hsa04360 | Axon guidance | 11 | 0.0425 | *PIK3R2, PLXNC1, PIK3CA, CFL2, SSH1, SEMA6D, EPHA2, EPHB2, PPP3CA, UNC5D, FZD3* |
| hsa04140 | Autophagy - animal | 10 | 0.0193 | *PIK3R2, STX17, PIK3CA, WDR41, TSC1, IRS1, PDPK1, PRKAA2, IRS2, MAPK8* |

**Table S2**. Enriched KEGG pathways of the deregulated EV-miRNA target genes.
